# A novel small deletion of *LMX1B* in a large Chinese family with nail-patella syndrome

**DOI:** 10.1186/s12881-019-0801-3

**Published:** 2019-05-03

**Authors:** Xiaoyi Yan, Jie Lin, Yifan Wang, Junli Xuan, Ping Yu, Tingwei Guo, Fan Jin

**Affiliations:** 1 Department of Cell Biology and Program in Molecular Cell Biology, Zhejiang University School of Medicine, Hangzhou, 310058 Zhejiang China; 2 The First Affiliated Hospital of Wenzhou Medical University, Wenzhou, Zhejiang, 325000 China; 3 Department of Orthopedics, Integrated Chinese and Western Medicine Hospital of Zhejiang Province, Hangzhou Red Cross Hospital, Hangzhou, 310003 Zhejiang China; 4 Imaging Facility of core facilities, Zhejiang University School of Medicine, Hangzhou, 310058 Zhejiang China; 5 Department of Genetics and Genomic Sciences, Icahn School of Medicine at Mount Sinai, New York, NY 10029 USA; 6 Department of Reproductive Endocrinology, Key Laboratory of Reproductive Genetics, Ministry of Education and Women’s Reproductive Health Laboratory of Zhejiang Province, Women’s Hospital School of Medicine Zhejiang University, Zhejiang, 310006 Hangzhou China

**Keywords:** Bioinformatic analysis, LIM homeobox transcription factor 1-beta (LMX1B), Mutation, Nail–patella syndrome, Sanger sequencing

## Abstract

**Background:**

Nail-patella syndrome (NPS) is an autosomal dominant developmental disorder most commonly characterized by dyplasia of nail or patella, the radial head or the humeral head hypoplasia, and, frequently ocular abnormalities and renal disease. It is caused by heterozygous loss-of-function mutations in the LMX1B gene, which encodes LIM homeodomain transcription factor and is essential for regulating the dorsal limb fate.

**Methods:**

A five generation pedigree was recruited. Genomic DNA was extracted from the peripheral blood samples. Mutation detection was performed by Sanger sequencing the LMX1B gene. In silico functional annotation of the variant was performed using the in silico predictors SIFT, PolyPhen-2 and Mutation Taster.

**Results:**

A novel heterozygous small deletion within exon 4 of LMX1B, c.712_714delTTC, was identified in a rare five-generation NPS pedigree. The mutation resulted in a deletion of the conserved amino acid phenylalanine at codon 238 (p.Phe238del), which located in the homeodomain of LMX1B may abolish DNA binding with the molecule. Conformational prediction showed that the variation could transform the helical structure comprising p.Phe234, p.Lys235, p.Ala236, and p.Ser237.

**Conclusion:**

We identified a novel NPS-causing LMX1B mutation and expanded the spectrum of mutations in the LMX1B gene. The c.712_714delTTC mutation may affect the quaternary structure of LMX1B, which is essential for the specification of dorsal limb fate at both zeugopodal and autopodal levels, leading to typical NPS.

## Background

Nail–patella syndrome (NPS, OMIM #161200) is a rare autosomal dominant disorder with an incidence of approximately 1 in 50,000 live births, involving the maldevelopment of elbow joints, nail hypoplasia, and absent or hypoplastic patellae [1]. It warrants particular attention because those affected progress to variable degrees of physical dysfunction involving different organs. Epidemiological studies have suggested that about 30–40% of patients with NPS develop a renal disease and open-angle glaucoma has been identified as a feature of the syndrome in some patients [2, 3].

Heterozygous loss-of-function mutations of *LMX1B* (*LIM homeobox transcription factor 1*, *beta*, NM_002316.3), a gene located on 9q33.3, have been widely accepted as the major pathogenic mechanism behind NPS. *LMX1B* comprises eight exons covering more than 90 kb and encodes the LIM homeobox transcription factor 1-beta protein, which consists of 395 amino acid residues. *LMX1B* is expressed in most human tissues, especially in thyroid, testis, skeletal muscle, and ocular and pancreatic islets [4–6]. Moreover, as an LIM homeodomain transcription factor, it is essential for regulating the dorsal limb fate. The molecule exhibits a protein structure containing three different types of domain: two cysteine-enriched LIM domains, zinc-binding domains at the N-terminus, a critical DNA-binding homeodomain consisting of 60 amino acids, and a C-terminal active sequence that is rich in glutamate and serine residues [1]. The majority of mutations were found in the LIM domain and homeodomain [7–12]. In this context, understanding the molecular mechanisms behind the actions of the *LMX1B* gene and the structure of the protein that encodes is significant to clarify the pathogenesis of NPS.

To date, heterozygous mutations causing NPS with severe symptoms have rarely been reported. In this paper, we document a large Chinese family affected by NPS possessing a novel small deletion of *LMX1B*.

## Methods

### Patients

**Fig. 1:**
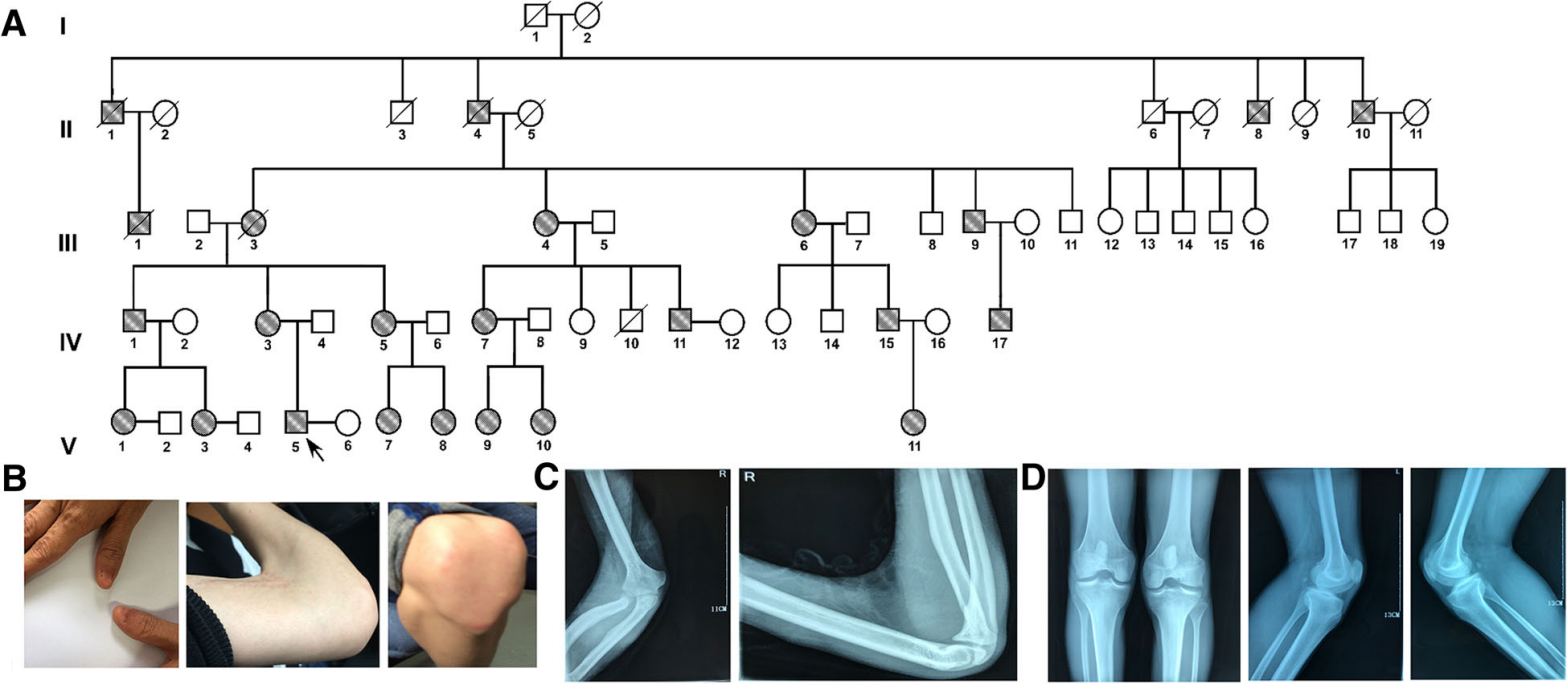
Pedigree of a Chinese family affected by nail–patella syndrome and the clinical phenotype of the patient V-5. **a** Pedigree of the Chinese family showing autosomal dominant inheritance of nail–patella syndrome. Dark solid squares (males) and solid circles (females) indicate affected individuals. Deceased individuals are indicated by a slash (/); the arrow shows the proband. **b** Clinical manifestations of the patient’s nails, elbows, and patellae. **c** Results of radiographic examination of the patient’s elbows. **d** Results of X-ray examination of the patient’s knee joint. The findings indicated smaller than usual bilateral patellae

A rare five-generation NPS pedigree from the Women’s Hospital of Zhejiang University School of Medicine was recruited. Samples from 11 affected and 10 unaffected individuals were collected (Fig. 1a).

### DNA extraction and qualification

Genomic DNA was extracted using the RelaxGene Blood DNA System (Tiangen, Beijing, China), in accordance with the manufacturer’s instructions. All DNA samples were dissolved in sterilized double-distilled water and stored at − 20 °C. DNA degradation was monitored on 1% agarose gels. All DNA samples were examined for protein contamination (as indicated by the A_260_/A_280_ ratio) and reagent contamination (indicated by the A_260_/A_230_ ratio) with a NanoDrop ND 1000 spectrophotometer (NanoDrop, Wilmington, DE, USA).

### Mutation detection

Seven pairs of primers flanking the regions of exons and exon–intron boundaries, including at least 60 bp from the exon–intron boundary of *LMX1B*, were designed by Primer3Plus (http://www.bioinformatics.nl/cgi-bin/primer3plus/primer3plus.cgi) and synthesized by Sangon Biotech Company (Shanghai, China) (Table 1). The PCR cycle conditions consisted of 5 min at 95 °C; 30 cycles of 30 s at 95 °C, 30 s at 58 °C, and 60 s at 72 °C; followed by 10 min at 72 °C. The potential variants were identified by Sanger sequencing using Big Dye terminator (Applied Biosystems, California, USA) on an automated DNA capillary sequencer (3700XL, Applied Biosystems).

**Table 1:** The primers for amplifying *LMX1B*

Primer	Sequence (5′- to 3′-)	PCR Product
LMX1B-Ex1-F	GCGTCCCATGGATATAGCA	258 bp
LMX1B-Ex1-R	ATGTCCTGCAAACCCATTTC	
LMX1B-Ex2-F	GAGGACTGGGACGGACTAGC	513 bp
LMX1B-Ex2-R	AGCTCTCGGAACCCTTGGAG	
LMX1B-Ex3-F	TTCTGCTGTCTGACCTGGTG	479 bp
LMX1B-Ex3-R	GAAGCAGGAGGTGGCTTCTC	
LMX1B-Ex4-F	GATCTTATCCTGGGCCACTG	474 bp
LMX1B-Ex4-R	TGGTGGGTTTAGGGGTATGA	
LMX1B-Ex5–6-F	ACCCCTAAACCCACCATCTC	566 bp
LMX1B-Ex5–6-R	CTTGGTGGAAGGCTTTTGAG	
LMX1B-Ex7-F	TTGGTAAGCTGAGCCTGGAG	547 bp
LMX1B-Ex7-R	ACAGGATGGCCTGCTGACTA	
LMX1B-Ex8-F	ACAGCCTACAGGGCAAACAG	405 bp
LMX1B-Ex8-R	TCTACCGGTCTGGCTGTACC	

### DNA subcloning

PCR products of exon 4 from patient V5 were subcloned into the pMD-19 T vector (TaKaRa, Dalian). Multiple independent colonies were picked and plasmids were extracted from subclones using the Qiagen Miniprep Plasmid Purification System according to the manufacturer’s instructions. Inserts were sequenced as described above to confirm the mutation.

### Bioinformatic analysis

The sequencing results were analyzed by Mutation Surveyor and aligned to the nucleotide sequence of *LMX1B* in the NCBI database. The pathogenicity of the novel mutation was evaluated using the in silico predictors SIFT (http://sift.jcvi.org/), PolyPhen-2 (http://genetics.bwh.harvard.edu/pph2/), and Mutation Taster (http://www.mutationtaster.org/). Models of the mutant protein were constructed by SWISS-MODEL. Variants were classified follows the guidelines and interpretation criteria established by the American College of Medical Genetics and Genomics (ACMG 2015) [13].

## Results

### Clinical manifestations and inheritance pattern

This study included 21 individuals from a single family, of whom 11 were affected by NPS. All individuals were examined for dysplastic nails of the thumbs, and hypoplastic patellae and elbows. Examinations showed that 73% (*n* = 8/11) of the patients suffered from nephropathy, while there was no ocular involvement in any patients. The proband (V-5) was a 27-year-old male, who had been diagnosed with NPS at the age of 7. Bilateral elbows showed clear positional abnormalities (Fig. 1b). Upon further X-ray examination of the proband, no significant changes in bone were found, joint space appeared to be normal, and there was no unusual soft tissue around the joints. Bilateral patellae were smaller than usual, while bilateral distal femoral and proximal tibial bones appeared to be normal. The articular surface was smooth, with no joint space stenosis. There was no joint capsule swelling and the infrapatellar fat pad was clear (Fig. 1c and d).

This five-generation family with NPS had 24 affected and 17 unaffected individuals in total. Among all of the affected subjects, there were 11 males and 13 females, and male-to-male transmission had occurred (IV-8 → V-17). These findings suggested the autosomal dominant inheritance of NPS.

### DNA subclone and sanger sequencing analysis

**Fig. 2:**
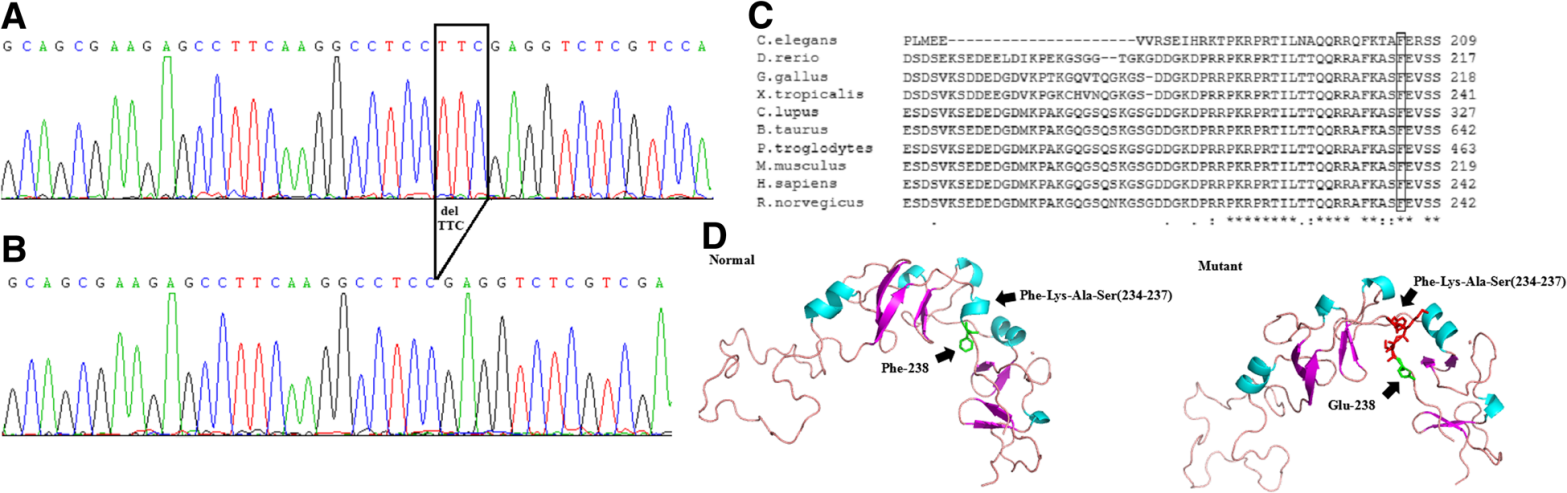
Sanger sequence and amino acid alignment of c.712_714delTTC of the *LMX1B* gene and prediction of the protein conformational changes by SWISS-MODEL. **a** Wild type allele DNA sequence of exon 4 of *LMX1B* (GenBank Accession: NM_002316.3) derived from subclone results of patient V-5. **b** The equivalent region from subclone results of patient V-5 showing the novel deletion mutation c.712_714delTTC. **c** Amino acid alignments show that c.712_714delTTC mutation occurs at a highly conserved position in *LMX1B*, as shown by comparing the corresponding sequence of 10 species. **d** Prediction of 3D structure changes in mutant LMX1B protein. The helical structure of Phe234-Lys235-Ala236-Ser237 was abolished and transformed into strand

The results of direct Sanger sequencing of patients revealed a heterozygous small in-frame deletion within exon 4 of *LMX1B* (NM_002316.3), c.712_714delTTC, which resulted in deletion of the amino acid phenylalanine at codon 238 (p.Phe238del). We also subcloned PCR products of exon 4 from patient V5, DNA sequencing of subcloned products confirmed this heterozygous mutation (Fig. 2a and b). This mutation is located in the homeobox (amino acid positions: 219–278 in NP_002307.2) of LMX1B. Using Sanger sequencing, it was confirmed in 10 other family members from whom DNA samples were available that the mutation cosegregated with the disease phenotype. Genetic analysis demonstrated that this mutation was carried by all affected patients (III-9, IV-1, 3, 5, 7, V-1, 3, 5, 7**–**9) but was absent from all healthy family members (III-10, IV-2, 4, 6, 8, 9, 14; V-2, 4, 6).

### In silico functional annotation of the variant

c.712_714delTTC was absent from the 1000 Genomes database, EVS, ExAC, and gnomAD (Genome Aggregation Database), which is considered a moderate piece of evidence for pathogenicity (PM2). This mutation was previously described as being of “uncertain significance” in ClinVar (RCV000624359.1, December 2017) by Ambry Genetics. One patient with this mutation having “inborn genetic diseases” was reported. Interestingly, mutation with the same amino acid code but different nucleotides (c.713 T > C; NP_002307.2: p.Phe238Ser) in this deletion region was reported as a mutation causative of NPS [7]. This same amino acid change evidence is considered strong support for a pathogenic interpretation for a rare Mendelian disorder (PS1).

The loss of phenylalanine located in the homeodomain, which is highly conserved in many species (Fig. 2c), probably impedes the DNA binding ability of the protein. The mutation was predicted to be damaging by three protein prediction algorithms: SIFT, PolyPhen-2, and Mutation Taster. This multiple lines of computational evidence support was be counted as supporting (PP3).

To predict the 3D structural changes in LMX1B proteins, we constructed the 3D structure of part of the protein that contains amino acids 52–241, and predicted 3D structural changes in LMX1B proteins. We found that Glu substitution could result in loss of the helical structure of Phe234-Lys235-Ala236-Ser237 into a strand, and this helix is a key region for DNA binding (Fig. 2d). Currently, the *LMX1B* gene mutation database (HGMD: http://www.hgmd.cf.ac.uk/ac/index.php) indicates that mutations most frequently occur in the homeodomain (positions 219–278), with nearly 30% of all known *LMX1B* mutations being clustered into this 60-amino-acid domain [9]. Thus, it is suggested that the occurrence of mutations in the homeodomain may be associated with a higher risk of NPS. It indicated that the occurrence of mutations in the homeodomain may be associated with a higher risk of NPS. Thus, this evidence can be considered moderate evidence of pathogenicity (PM1). Therefore, this variant with evidence of 1 PS (PS1), two PM (PM1 and PM2) and 1 PP (PP3) were considered to be likely pathogenic for NPS according the guidelines and interpretation criteria established by the American College of Medical Genetics and Genomics (ACMG 2015).

## Discussion

LMX1B is a member of the LIM homeodomain protein family characterized by the presence of two zinc-binding LIM domains and a homeodomain, which is essential for the specification of dorsal limb fate at the zeugopodal and autopodal levels in vertebrates [14]. The two LIM domains are characterized by a common cysteine-rich zinc-binding motif and are important in mediating protein–protein interactions, while the homeodomain of LMX1B is known to bind the FLAT element and is responsible for binding to DNA [15]. Various studies on the biological processes associated with LMX1B have been performed. In embryonic development, the *LMX1B* gene plays a pivotal role in limb development, kidney morphogenesis, and development of the anterior segment of the eyes [16].

This paper reports a five-generation NPS pedigree. The phenotypes of NPS in this family were consistent with those reported in the literature. The proband primarily manifested with nail-bed shortening and longitudinal ridging. In addition, typical malformations were clearly visible in the proband’s elbows and patellae (Fig. 1b–d). He had also developed nephropathy at the age of 22. Overall, 8 of the examined 11 patients presented renal involvement. The lack of phenotypic similarity between patients is not surprising given that the phenotype of this disease is extremely variable both within and between families. Another potential explanation for this lack is that the occurrence of renal disease is age-related, since the three patients (V-7, V-8, and V-9) free of a renal phenotype were younger than the others. One novel disease-causing mutation of *LMX1B* was identified in this study. This variant was absent from public databases and from the healthy individuals, and was predicted to be damaging by three protein prediction algorithms. In addition, a mutation associated with the same amino acid code but produced by different nucleotides was reported to be causative of the same disease [6]. To date, two pathogenic in-frame deletion mutations in other sites of *LMX1B* gene have been reported. Hamlington et al. reported a three-nucleotides in-frame deletion mutations within the conserved homeodomain [9] and Clough et al. repported a nine-nucleotides in-frame deletion mutations within LIM domain [17]. Therefore, the variant identified here was predicted to be likely pathogenic for NPS.

## Conclusions

In conclusion, our study identified a novel in-frame deletion of *LMX1B*, c.712_714delTTC (p.Phe238del), which could cause an aberrant LMX1B protein conformation, as a likely pathogenic factor causative of NPS. Prenatal DNA diagnosis and genetic counseling should be provided to families possessing this variant.

## Abbreviations

HGMD: Human Gene Mutation Database
LIM: Lin-1, Isl-1 and Mec-3
LMX1B: LIM homeobox transcription factor 1-beta
NPS: Nail-patella syndrome
PCR: Polymerase chain reaction
SIFT: Sorting Tolerant From Intolerant
